# Distribution Clearance: Significance and Underlying Mechanisms

**DOI:** 10.1007/s11095-024-03738-7

**Published:** 2024-07-09

**Authors:** Michael Weiss

**Affiliations:** Department of Pharmacology, Martin Luther University Halle-Wittenberg, Magdeburger Straße 20 (Saale), 06112 Halle, Germany

**Keywords:** compartmental model, distribution clearance, distribution kinetics, permeability-surface product, pharmacokinetic analysis

## Abstract

**Purpose:**

Evaluation of distribution kinetics is a neglected aspect of pharmacokinetics. This study examines the utility of the model-independent parameter whole body distribution clearance (*CL*_*D*_) in this respect.

**Methods:**

Since mammillary compartmental models are widely used, *CL*_*D*_ was calculated in terms of parameters of this model for 15 drugs. The underlying distribution processes were explored by assessment of relationships to pharmacokinetic parameters and covariates.

**Results:**

The model-independence of the definition of the parameter *CL*_*D*_ allowed a comparison of distributional properties of different drugs and provided physiological insight. Significant changes in *CL*_*D*_ were observed as a result of drug-drug interactions, transporter polymorphisms and a diseased state.

**Conclusion:**

Total distribution clearance *CL*_*D*_ is a useful parameter to evaluate distribution kinetics of drugs. Its estimation as an adjunct to the model-independent parameters clearance and steady-state volume of distribution is advocated.

## Introduction

Total distribution clearance (*CL*_*D*_) is defined as measure of distribution kinetics of drugs in the body that is independent of a specific structural model. In order to segregate the distribution from the elimination process, it is based on the area under the curve in a hypothetical noneliminating system [1]. Due to this clear interpretability, the parameter distribution clearance can be estimated from drug disposition data using compartmental models, physiologically-based pharmacokinetic (PBPK) models or circulatory models (based on transit time densities) [2], and therefore it allows a systematic comparison of drugs with respect to their distributional properties. Together with elimination clearance *CL*_*D*_ quantifies the extent of deviation of drug disposition curves from monoexponential disposition.

Here the distribution clearances of 15 drugs were calculated and the underlying mechanisms were investigated. The results of this review show that additional useful information and physiological insight can be gained by estimating *CL*_*D*_. That the evaluation of distribution kinetics, ie of the rate of drug distribution, is a neglected feature of pharmacokinetics was already pointed out by Atkinson [3].The objective for the present analyses was to demonstrate the utility of the parameter *CL*_*D*_ for this purpose.

## Methods

### Noneliminating System

**Fig. 1:**
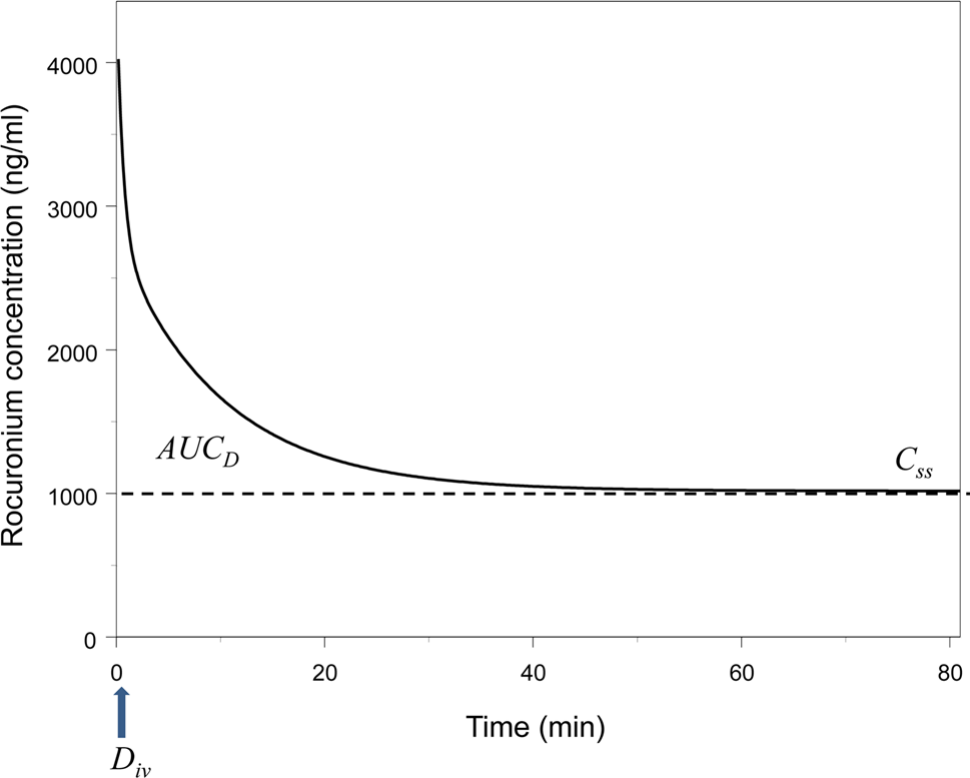
The area *AUC*_*D*_ between the concentration–time curve and the steady state concentration *C*_*ss*_ in a noneliminating system The curve was simulated using the population mean parameter estimates for rocuronium [2] setting *CL* = 0

The parameter distribution clearance is determined by the area under concentration–time curve (*AUC*_*D*_) in a hypothetical noneliminating (closed) system (*CL* = 0), that means the area between *C*_*D*_(*t*) after bolus injection of dose *D*_*iv*_ and the concentration reached at steady state.

$$\left({C}_{SS}={D}_{iv}/{V}_{SS}\right)$$ (Fig. 1) [1]:

1 $${CL}_{D}=\frac{{D}_{iv}}{{AUC}_{D}}$$

The *C*_*D*_(*t*) curve (Fig. 1) reflects what “kinetics” means in a physical context: the tendency of a system to reach a state of equilibrium.

The measure *CL*_*D*_ can be calculated from the parameters of different pharmacokinetics models, as mammillary compartment models, sum of exponentials, PBPK models and recirculatory models [2]. Since compartmental models (Eq. 6) or sums of exponentials (Eq. 8) are commonly used in practice for analyzing drug disposition data, these models were applied in the present study to calculate ${CL}_{D}$, despite their failure to describe the initial distribution (see Limitations below). Note that Eqs. 6, 7, 8, 9, 10, 11, 12, 13, 14, 15 and 16 were moved to Appendix in order to preserve the readability of the text.

The parameter ${CL}_{D}$ was originally defined by Eq. 1 in terms of a recirculatory model. Later it became clear that a definition based on mass transfer out of the initial distribution volume ${V}_{0}$ (where the drug distributes instantaneously at *t* = 0) may be more appropriate from a physical point of view. The distribution process can be described by

2 $$\frac{{dA}_{0}\left(t\right)}{dt}=-{CL}_{D}\left[{C}_{D}\left(t\right)-{C}_{SS}\right]$$

where ${A}_{0}\left(t\right)={V}_{0}{C}_{D}\left(0\right)$ is the amount of drug in ${V}_{0}={D}_{iv}/{C}_{D}\left(0\right)$. After integrating both sides of Eq. 2, we obtain ${CL}_{D}$ as

3 $${CL}_{D,corr}=\frac{{D}_{iv}\left(1-{V}_{0}/{V}_{SS}\right)}{{AUC}_{D}}$$

ie ${CL}_{D,corr}=\left(1-{V}_{0}/{V}_{SS}\right){CL}_{D}$. Since in all previous publications ${CL}_{D}$ (also called ${CL}_{M}$) was calculated by Eq. 1, the term ${CL}_{D,corr}$ will be used for the definition by Eq. 3.

To this end, based on the individual parameters of compartment models $\left({V}_{0}, {V}_{1}, {V}_{2},{CL}_{02}{,CL}_{02}\right)$ and sum of exponentials $\left({B}_{i}, {\lambda }_{i}, i=1..3\right)$ the distribution clearances were calculated using Eq. 6 and Eq. 8, respectively. Apart from parameters estimated in our own studies, parameters from the literature were employed. The selection of drugs was somewhat arbitrary since only those publications could be considered where the individual parameter estimates were available. Furthermore, only results obtained with a three-compartment or a triexponential model were selected. Here we report the means and coefficients of variations of the ${CL}_{D}$ values of the drugs. Note that in contrast to the sum intercompartmental clearances ${CL}_{0i}$ between central volume ${V}_{0}$ and volumes ${V}_{i},\sum_{i=1}^{n}{CL}_{0i}$ (eg Ref. [4]), which characterizes only the initial distribution process at time t = 0 [5, 6], *CL*_*D*_ describes the overall distribution behavior in the body.

Linear regression analysis with pharmacokinetic parameters and available covariates were performed to reveal information on which factors determine the distribution process. For comparison with the conventionally used empirical measure ${CL}_{D}$, the physically more realistic parameter *CL*_*D,corr*_ (Eq. 3) as also reported.

### Eliminating System

**Fig. 2:**
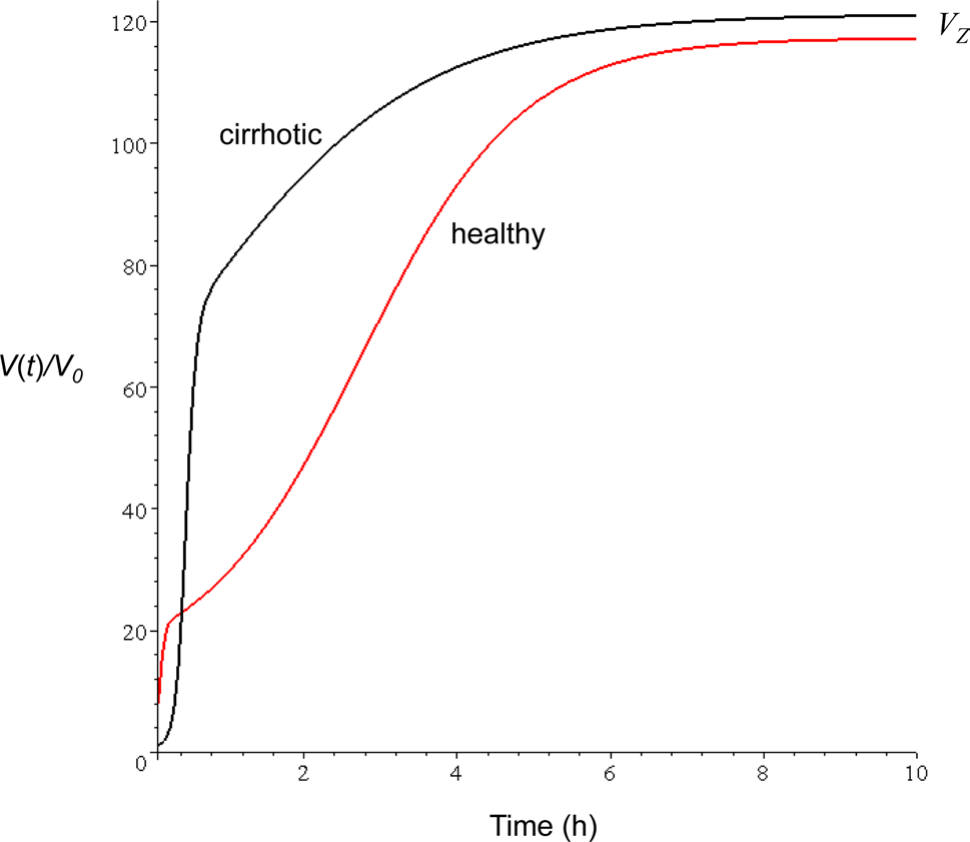
Time dependent fractional volume of distribution (Eq. 5) for chlormethiazole in healthy volunteers (*CL*_*D*_ = 0.8) and cirrhotic patients (*CL*_*D*_ = 3.1).The curves were simulated using the pharmacokinetic parameters published in Ref. [9]

To separate distribution and elimination process, ${CL}_{D}$ was defined above by setting the elimination clearance, $CL=0$. In the real eliminating (open) system ${CL}_{D}$ determines the departure from the one-compartment behavior (monoexponential decay of the disposition curve). A measure of this deviation is given by [7]

4 $${RD}_{D}^{2}-1=\frac{2CL}{{CL}_{D}}$$

where ${RD}_{D}^{2}$ denotes the relative dispersion (normalized variance) of the disposition residence time distribution (Eq. 11). For an instantaneous distribution in the body, ie ${CL}_{D}\to \infty$, one gets ${RD}_{D}^{2}-1\to 0$. The role of the measure (Eq. 4) characterizing the effect of the distribution kinetics can be best demonstrated by defining a time-varying volume of distribution according to

5 $$V\left(t\right)=\frac{A\left(t\right)}{C\left(t\right)}$$

where *A*(*t*) and *C*(*t*) are the drug amount in the body and plasma concentration after bolus injection. *V*(*t*) was proposed by Niazi [8], who calculated its time course for a multiexponential drug disposition curve. As an example Fig. 2 shows the time courses of *V*(*t*) calculated from the parameters published for chlormethiazole in healthy volunteers and patients with cirrhosis of the liver [9], that are characterized by 2*CL/CL*_*D*_ values of 1.0 and 4.6, respectively. The time courses of *V*(*t*) clearly shows the effect of slow and rapid distribution (*CL*_*D*_ = 0.8 and 3.08). (The origin of the difference in *CL*_*D*_ will be discussed below.) The increase in *V*(*t*) reflects the evolution towards a thermodynamic equilibrium state in the distribution process where the equilibrium volume *V*_*z*_ is reached asymptotically. However, in contrast to the distribution process in the closed system (Fig. 1) where the static equilibrium is characterized by *V*_*ss*_ (*C*_*ss*_ = const.), *V*_*Z*_ denotes a dynamic equilibrium (*A*(*t*)/*C*(*t*) = const.). The ratio *V*_*Z*_*/V*_*ss*_ increases in parallel to ${RD}_{D}^{2}$ and generally the following relationship holds [7]:

$${V}_{0}\le {V}_{SS}\le {V}_{Z}$$

with equality in the limiting case of instantaneous distribution at *t* = 0.

Since a one-compartment disposition model (Bateman function) is often used for oral data, one can expect that in this case a better fit is obtained for drugs with a low 2*CL/CL*_*D*_ value (especially if the absorption rate is relatively high).

## Results and Discussion

The results are summarized in Table I where the means and coefficients of variation of ${CL}_{D}$ as well as of ${CL}_{D,corr}$ are reported together with the measure 2*CL/C*_*D*_ for the eliminating system. Furthermore, the results of linear regression analysis are presented indicating the relationship to pharmacokinetic parameters and contribution of covariates to ${CL}_{D}$. Note that these correlations are of empirical nature. Particularly striking is the difference between the ${CL}_{D}$ values of 0.19 L/min for gadoxetate and of 6.64 L/min for propranolol, which is connected with the highest and lowest deviation from a monoexponential disposition curve, respectively (with 2*CL/CL*_*D*_ values of 15.9 and 0.5). Significant changes in ${CL}_{D}$ were observed under certain conditions for gadoxetate [13], talinolol [14], thiopental [16] and chlormethiazole [9].

**Table I:** Total Distribution Clearance (*CL*_*D*_) with Intersubject Variability (CV) and Relationship to Parameters and Covariates

Drug	*CL*_*D*_	CV	Subjects, n	Condition	Linear	Slope	R	p value	*CL*_*D,corr*_	CV	2*CL/CL*_*D*_^a^	Ref.^b^
	L/min	%			regression				L/min	%		
Rocuronium	0.628	43	Patients, 10		CL_D_ vs f_u_PS	1.5	0.89	< 0.001	0.509	38	1.6	[2, 10]
					CL_D_ vs CO	0.11	0.7	< 0.05				
Trospium	0.593	23	healthy, 12		CL_D_ vs Vss	0.002	0.8	< 0.01	0.57	24	3.2	[11]
Propiverine	0.829	40	healthy, 10		CL_D_ vs CL	0.01	0.88	< 0.001	0.384	40	.58	[12]
					CL_D_ vs Vss	0.01	0.9	< 0.001				
Gadoxetate	0.039	64	healthy, 12	OATP *1a/*1a					0.027		8.6	[13]
	0.019	50	healthy, 9	OATP *15/*15, p < 0.01	CL_D_ vs kin/kou^c^	1.3	0.77	< 0.001	0.014		15.9	
Talinolol	0.796	8	healthy, 7	Control							.81	[14]
	1.1	23		with Rifampicin, p < 0.01	∆CL_D_ vs ∆CL^d^	2.95	0.79	< 0.05			.81	
R-Ketamine	3.43	44	healthy, 15		CL_D_ vs Vss	0.002	0.58	< 0.05	3.21	32	1.6	[15]
Thiopental	0.902	7	Patients, 7	Control	CL_D_ vs Vss	0.003	0.55	< 0.05			0.45	[16]
	0.573	26	Patients, 7	with Dexmet.^e^, p < 0.0001							0.65	
Fentanyl	3.95	84	healthy, 5								0.49	[17]
Alfentanil	0.863	41	healthy, 5		CL_D_ vs CL	0.08	0.95	< 0.05			0.46	[17]
Alfentanil	1.21	40	Patients, 7		CL_D_ vs Q	0.26	0.89	< 0.05	1.12	43	0.56	[4]
					CL_D_ vs TBW	0.03	0.89	< 0.01				
Sufentanil	1.53	36	Patients, 10						1.47	35	1.4	[18]
Chlormethiazole	0.82	30	healthy, 6	Controls	CL_D_ vs ALB^f^	-2.51	0.66	< 0.05	0.759	25	4.6	[9]
	3.08	40	Patients, 8	Cirrhosis, p < 0.01					2.84	33	1.0	
Acebutalol	1.89	32	healthy, 5						1.68	30	0.69	[19]
Propranolol	6.64	62	healthy, 6						5.76	62	.50	[20]
Vancomycin	0.623	36	Patients, 6	Obese	CL_D_ vs CL	1.87	0.69	p < 0.05	0.566	39	0.63	[21]
	0.445	34	healthy, 4	Normal	CL_D_ vs Vss	0.01	0.64	p < 0.05	0.381	37	0.37	
					CL_D_ vs TBW	0.002	0.69	p < 0.05				
Digoxin	0.74	16	healthy, 5						0.696	14	0.35	[22]

^a^ Measure of the deviation from a pure exponential disposition curve (Eq. 4)

^b^ References from which individual parameters of 3-compartment or triexponential model were obtained

^c^ Ratio of hepatic uptake to efflux constant

^c^ ∆: Value with rifampicin minus control value

^e^ Dexmedetomidine

^f^ Serum albumin level

In order to understand the differences between the estimated *CL*_*D*_ values of the 15 drugs in Table I, we have to identify potential influencing factors of the distribution process.

### Capillary Permeability

**Fig. 3:**
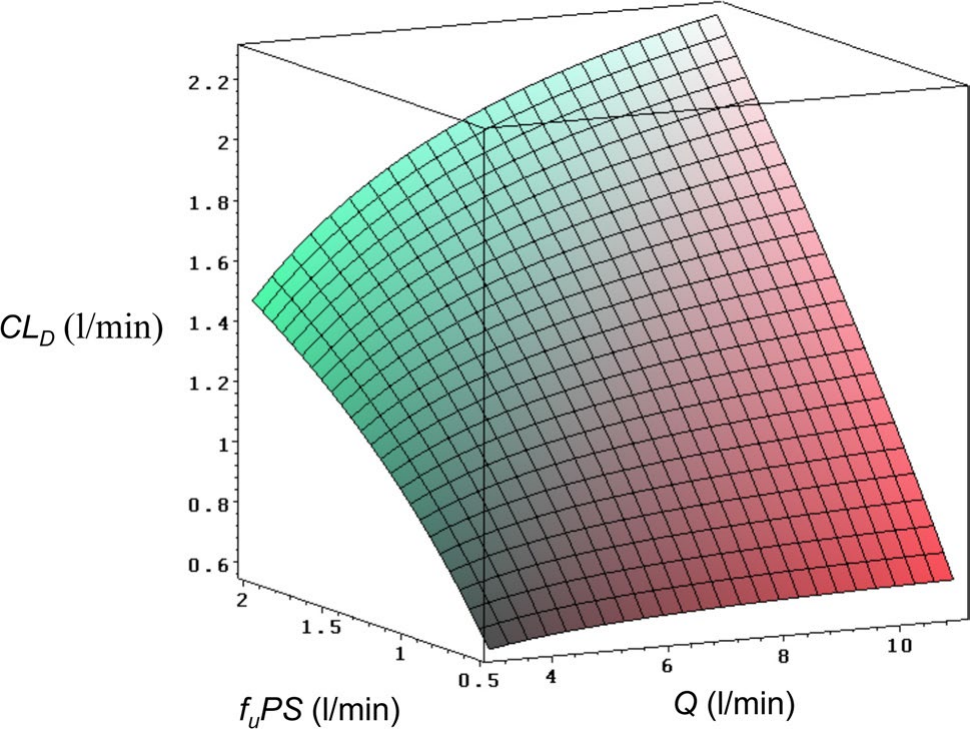
Permeability-surface area product (*f*_*u*_*PS*) and cardiac output (*Q*) as determinants of distribution clearance (*CL*_*D*_). The plot was calculated by Eqs. 13 and 14 assuming ${RD}_{B}^{2}$ = 3, *V*_*B*_ = 5 l and *V*_*T*_ = 25 l. The volume parameters are those of alfentanil [4]

Generally mass transfer out of the vascular space is limited by a permeability barrier. Thus apart from the unbound fraction (*f*_*u*_) discussed below, ${CL}_{D}$ is determined by the permeability-surface area product (*PS*) and blood flow. The prediction obtained from the recirculation model (organs of the systemic circulation lumped in a single subsystem, see Eqs. 13 and 14) was visualized for alfentanil by a response surface plot in order to illustrate the effect of tissue permeability (*f*_*u*_*PS*) and cardiac output (*Q*) on *CL*_*D*_ (Fig. 3). Note that in this simplest model of the systemic circulation the parameters, *V*_*B*_*,V*_*T*_ and *f*_*u*_*PS* are the apparent parameters for the systemic circulation averaged over all organs. Figure 3 shows that *CL*_*D*_ is mainly determined by *f*_*u*_*PS*, and it becomes nearly independent of *Q* for low values of *f*_*u*_*PS*. Although the increase of *CL*_*D*_ with *Q* observed for alfentanil (Table I), is in principal accordance with the prediction of the simplified recirculation model (assuming ${RD}_{B}^{2}$ = 3 [23]), the present compartmental approach does not properly describe the flow dependency, since the effect of initial distribution is neglected (see discussion below). Note that based on estimating the relative dispersion of the circulatory transit time distribution ${RD}_{C}^{2}$ with a circulatory model, inulin and antipyrine were characterized by barrier-limited and perfusion-limited distribution, respectively [23]. Thus the distribution of alfentanil presents an intermediate situation between these two extremes.

Since plasma protein binding controls the free drug concentration in plasma, and only unbound drug molecules can cross the membrane barrier, it is a main determinant of distribution clearance. This is demonstrated by the significant increase of distribution clearance of chlormethiazole in patients with cirrhosis (from 0.82 to 3.05 L/min) (Table I). This may be mainly attributed to the increase in the free fraction *f*_*u*_ as result of the decrease in serum albumin. First, *f*_*u*_ was statistically significantly increased in patients with cirrhosis by 33% [9]; and second, *CL*_*D*_ was negatively correlated with the serum albumin level (R = 0.66, p < 0.05), which is important in the light of the significant correlation of serum level of albumin and percentage chlormethiazole bound to plasma proteins [9]. The effect of the higher free fraction of drug in cirrhotic patients on the time course of *V*(*t*), ie the more rapid distribution, is shown in Fig. 2.

One may speculate that the significant positive correlation between *CL*_*D*_ and *V*_*ss*_ or *CL* found for some of the drugs (trospium, propiverine, R-ketamine and vancomycin) could be explained by inter-individual differences in the serum albumin levels, since both *CL*_*D*_ and *V*_*ss*_ increase with the fraction unbound. An effect of free fraction on inter-subject variability in distribution was also suggested by Upton et al. [24] (due to variability in albumin concentration and/or binding affinity). For alfentanil, for example, an inter-individiual variability in *f*_*u*_ of 42% was observed [25]. More obvious is the role of total body weight (*TBW*) in this respect. For vancomycin, a significant correlation was found between between *CL*_*D*_ and *TBW* [21]. In this case the positive correlations between *CL*_*D*_ and *V*_*ss*_ as well as between *CL*_*D*_ and *CL*, can be explained by significant correlations between *V*_*ss*_ and *TBW* (R = 0.89, p < 0.01) and between *CL* and *TBW* (R = 0.94, p < 0.01). That also for alfentanil [4] *CL*_*D*_ correlates with *TBW* indicates that part of the inter-individual variability in *CL*_*D*_ is dues to the variability in *TBW*.

Interestingly, no correlation between *CL*_*D*_ and lipophilicity (log P) was observed. This may be due to the role of plasma protein binding as a cofounding factor. If the degree of binding is similar as for thiopental and fentanyl (about 70%) [26], the higher lipophilicty of fentanyl (Log P: 3.89) compared to thiopental (Log P: 1.85) [27] explains higher *CL*_*D*_ (3.95 vs. 0.90 l/min). That the *CL*_*D*_ of alfentanil (1.21 l/min) is lower than that of fentanyl, on the other hand, may be caused by both a higher protein binding (about 90% [26]) and a lower lipophilicity (log P: 2.16) [27]. Note also that the ratio of *CL*_*D*_ of alfentanil to that of fentanyl (0.31) is quite similar to the ratio of uptake clearances of alfentanil and fentanyl measured in rat muscle (0.44) [28].

### Cardiac Output

For rocuronium and alfentanil where an independent estimate of cardiac output was available, a significant correlation between *CL*_*D*_ and *Q* was found (Table I). However, in these cases *CL*_*D*_ increases only moderately with *Q* (with slopes between 0.11 for rocuronium and 0.26 for alfentanil). However, even for highly permeable drugs, the distribution out of the vascular space occurs not instantaneously and flow-limited distribution $\left({RD}_{C}^{2}\to {RD}_{B}^{2 }\ \mathrm{for}\ {f}_{u}PS\to \infty \right)$, Eq. 14) is a theoretical limiting case for which *CL*_*D*_ cannot be defined. Interestingly, the *CL*_*D*_/*Q* ratio of 0.25 predicted by Eqns. 13 and 14 for alfentanil (*Q* = 6 l/min, *f*_*u*_*PS* = 1.4 l/min) is quite similar to the ratio of 0.2 calculated for thiopental [29]. That the *CL*_*D*_ of thiopental of 0.902 L/min was significantly decreased by 40% in patients treated with the α_2_-adrenoceptor agonist dexmedetomidine may be attributed to a decrease and redistribution of cardiac output [14]. This is similar to the result calculated form the parameters reported in a porcine model of hemorrhagic shock [30], where the reduction in *CL*_*D*_ of fentanyl from 2.99 ± 0.97 L/min to 1.91 ± 0.82 L/min in the shock group was likely due to a redistribution of cardiac output accompanied by the decrease in cardiac index by 43%.

In discussion the hemodynamic influences on *CL*_*D*_ using Eq. 13, we have to consider the neglected the effect of regional distribution of cardiac output (organ blood flows) and the heterogeneity of distributional properties of organs, which affect the relative dispersion of circulation times,${RD}_{C}^{2}$ (Eq. 14) and therefore *CL*_*D*_ (Eq. 13). This means in applying Eq. 14 a compromise was made between necessary simplifications and the aim to reveal the main influencing factors of *CL*_*D*_*.* At this point the relationship to PBPK models (eg Refs. [28, 31, 32]) should be discussed. Assuming a multi-organ model where the *i* organs/tissues of the systemic circulation (with blood flows *Q*_*i*_) are described by well-mixed vascular and tissue compartments( distribution volumes *V*_*B*i_ and *V*_*Ti*_), separated by a permeability barrier (*f*_*u*_*PS*_*i*_), *CL*_*D*_ can be easily defined in terms of these parameters (in analogy to the case of well-mixed organs (Eq. 15)) [33]. But although the distribution clearance could be more accurately predicted using a PBPK model, this approach is not practicable in the present context due to the large number of unknown parameters. Note also that the ${RD}_{C}^{2}$ predicted for an intravascular marker (Eq. 15) shows that not *Q* per se but a redistribution of blood flow affects ${RD}_{C}^{2}$.

A feasible approach, however, could be the reduction of the systemic circulation into two heterogeneous subsystems (Eq. 17 with *n* = 2). For the lipophilic drug thiopental, for example, a splitting into fat and non-fat tissues improved the fit and allowed a prediction of *CL*_*D*_ as a function of percentage body fat in obese subjects [29].The predicted *CL*_*D*_*/Q* ratio of 0.2 in lean subjects is in accordance with the estimate reported in Table I.

### Active Transport

Of particular note is the fact that the distribution clearance of gadoxetate is about one magnitude lower than those of the other compounds (Table I). It takes almost 40 h until distributional equilibrium is achieved (Fig. 3 in Ref. [13]) compared to about 1 h for rocuronium (Fig. 1). The reason is that gadoxetate leaves the vascular space primarily by uptake into hepatocytes whereas extravascular permeation is relatively low in comparison to the other compounds [13]. Gadoxetate is transported into hepatocytes by the organic anion transporting polypeptide (OATP) 1B1, OATP1B3, and backflux into the sinusoids occurs via the transport protein MRP3 and/or bidirectional-acting OATPs. Since the efflux from hepatocytes is slower than the uptake, gadoxetate accumulates in the liver. The finding that *CL*_*D*_ of gadoxetate in carriers of the variant OATP1B1*15/*15 was significantly smaller than in carriers of the wild-type protein, OATP1B1*1a/*1a could be explained by an increased sinusoidal efflux rate in subjects with the variant *15/*15 protein compared with the wild type *1a/*1a. This corresponds to the linear increase in *CL*_*D*_ with the ratio of hepatic uptake to efflux constant as well as with the tissue-to-plasma transport constant estimated with the PBPK model. The *fuPS* value of gadoxetate was ten-fold lower than that of rocuronium [10].

Another example where active transport may play a role in distribution kinetics is talinolol, where *CL*_*D*_ increased significantly under rifampicin treatment (Table I). Since talinolol is a substrate of the efflux transporter P-glycoprotein (P-gp), this suggests that rifampicin-mediated P-gp induction leads to an increase in *CL*_*D*_. Furthermore, this increase correlated with the increase in elimination clearance (R = 0.79, p < 0.05), which has been attributed to an increase of Pgp mediated intestinal secretion [34, 35]. Although no clear explanation is available for the increase in *CL*_*D*_ as a consequence of rifampicin-mediated P-gp induction, it could be due to an intestinal reabsorption of talinolol.

### Slow Tissue Binding

An interesting special case is the distribution kinetics of digoxin. The high distribution volume of digoxin of about 600 l is mainly determined by binding to skeletal muscular Na^+^/K^+^-ATPase (sodium pumps) at the extracellular side of the plasma membrane [36]. Digoxin permeates into the interstitial space (with negligible cellular uptake), but although the distribution is not diffusion limited with a *fuPS* value that is about 20-fold higher than that of rocuronium [10], their *CL*_*D*_ values are quite similar. This unexpected low distribution clearance of digoxin is primarily determined by slow tissue binding (mainly to skeletal muscle), ie binding of digoxin molecules to receptors (sodium pumps, Na^+^/K^+^-ATPase) at the extracellular side of the plasma membrane. Pharmacokinetic modeling predicts a time constant of 34 min for binding equilibration and suggests that a ~ 1.5 fold increase in digoxin binding leads to a ~ 20% increase in *CL*_*D*_ [22]. This corresponds to the finding that stimulation of the Na^+^/K^+^-pump in skeletal muscle by the β_2_-agonist salbutamol affects distribution kinetics of digoxin in a similar fashion [37].

### Limitations

In this study, as mostly in pharmacokinetics, drug disposition curves are regarded as decreasing (ie ${RD}_{D}^{2}$ > 1 in Eq. 11 and ${RD}_{C}^{2}$ > 1 in Eq. 13). The implicit assumption of an instantaneous distribution into an initial distribution volume *V*_*0*_ (volume of the central compartment, see also Eq. 10) is a limitation in the estimation and interpretation of *CL*_*D*_ values. Assuming that initial distribution is already complete (*C*(0) = *D*_*iv*_/*V*_*0*_), rapid distribution within the first minutes after bolus injection is not covered by the present approach. Thus it cannot be applied to highly permeable drugs like antipyrine with flow-limited distribution kinetics where *CL*_*D*_ is mainly determined by the initial distribution phase. One approach to deal with initial distribution is recirculatory modeling based on frequent early arterial blood sampling (within the first 3 min) and the simultaneous injection of an intravascular marker distribution (like indocyanine green, ICG). That the estimate of ${RD}_{C}^{2}$ for antipyrine is then only slightly higher than that for ICG (characterizing intravascular mixing) [23], indicates that antipyrine distributes in the whole body similarly fast like ICG in the vascular space. Note that the circulatory mixing time of an intravascular indicator increases with ${RD}_{C}^{2}$ and decreases with *Q* [38]. The assessment of early drug distribution is of special importance for intravenously administered anesthetic drugs [39] and circulatory models have a high heuristic value in explaining the effect of drug interactions and hemodynamic changes (eg [5, 23, 40]).

It should be further noted that also in the present case of decreasing *C*(*t*) curves (compartmental modeling), the estimated *V*_*0*_ may depend on the early sampling schedule. If sampling starts relatively late or if a 2-compartment model is applied, unrealistically high *V*_*0*_ values may be obtained. In this case (when *V*_*0*_ is not much smaller than *V*_*ss*_), *CL*_*D*_ values higher than cardiac output can be obtained, while theoretically *CL*_*D*_ ≤ *Q* (Eq. 13). This was not the case in the present examples where a 3-compartment model or a sum of three exponentials was applied, and it would not appear if distribution kinetics is assessed by *CL*_*D,corr*_. Generally, however, the *CL*_*D,corr*_ values were not much different from those of *CL*_*D*_; with the exception of drugs like propiverine with a ratio of *V*_*0*_/*V*_*ss*_ of about 0.5.

Thus, although the definition of *CL*_*D*_ is independent of a specific structural model, the limitations inherent in its estimation and explanation (eg in terms of a mechanistic model) should be always taken into account. Both are dependent on the underlying assumptions. Finally note also that our model is based on system linearity (as assumed in the underlying studies).

## Summary

The results demonstrate that the distribution clearance *CL*_*D*_ is a useful measure of distribution kinetics of drugs. It enables a comparison of the distributional properties of different drugs and reflects the effect of drug-drug interactions, transporter polymorphisms and diseased states. The estimation of *CL*_*D*_ offers physiological insights due to its interpretability by mechanistic models. *CL*_*D*_ could serve as a sensitive parameter to characterize intersubject variability of distribution kinetics and analysis of covariates may reveal influencing factors. The physiological interpretability of *CL*_*D*_ makes this parameter useful in drug development. It is hoped that this study will stimulate the estimation of *CL*_*D*_ as an adjunct to the other model-independent pharmacokinetic parameters *CL* and *V*_*ss*_. For a comprehensive review of mechanisms of drug distribution and the relationship between the drug distribution and pharmacologic response, see eg Ref. [41].

## Appendix

### Mammillary Compartment Model

The distribution clearance for a mammillary compartment model with peripheral compartment volumes *V*_*i*_ and intercompartmental clearances *CL*_*0i*_ (between central volume *V*_*0*_ and volumes *V*_*i*_) is given by [6]:

6 $${CL}_{D}={\left[\sum_{i=1}^{n}{\left(\frac{{V}_{i}}{V_{ss}}\right)}^{2}\frac{1}{{CL}_{0i}}\right]}^{-1}$$

### Sum of Exponentials

If the disposition data were fitted by a sum of exponentials

7 $$C\left(t\right)=\sum_{i=1}^{n}{B}_{i}{e}^{-{\lambda }_{i}t}$$

we have [1]

8 $${CL}_{D}=CL\left[\frac{\sum\limits_{i=1}^{n}\left(\frac{{B}_{i}}{{\lambda }_{i}^{3}}\right)\sum\limits_{i=1}^{n}\left(\frac{{B}_{i}}{{\lambda }_{i}}\right)}{{\left(\sum\limits_{i=1}^{n}\frac{{B}_{i}}{{\lambda }_{i}}\right)}^{2}}-1\right]$$

where

9 $$CL=\frac{{D}_{iv}}{\sum\limits_{i=1}^{n}\frac{{B}_{i}}{{\lambda }_{i}}}$$

and the initial distribution volume is given by

10 $${V}_{0}=\frac{{D}_{iv}}{\sum\limits_{i=1}^{n}{B}_{i}}$$

### Relative Dispersion of Disposition Residence Time Distribution

As proved in Ref. [7], the relative dispersion (normalized variance) of disposition residence time distribution, ${RD}_{D}^{2}$, determines the deviation from the one-compartment behavior or monoexponential disposition curve (ie instantaneous distribution in the body) where ${RD}_{D}^{2}$ = 1 and the deviation is given by ${RD}_{D}^{2}-1$, ie for decreasing drug disposition curves ${RD}_{D}^{2}>1$ holds.

Furthermore one can show that [1]

11 $${CL}_{D}=\frac{2CL}{{RD}_{D}^{2}-1}$$

Thus in terms of ${CL}_{D}$ and *CL*, a measure for deviation from the one-compartment behavior is given by.

12 $$\frac{2CL}{{CL}_{D}}$$

The whole body distribution clearance ${CL}_{D}$ should not confused with the organ distribution clearance defined in physiological base pharmacokinetic (PBPK) models [28, 42].

### Recirculatory model

For a recirculatory model with cardiac output *Q* and relative dispersion of the circulatory transit time distribution ${RD}_{C}^{2}$ one gets [1]

13 $${CL}_{D}=\frac{2Q}{{RD}_{C}^{2}-1}$$

Applying an equation that has been derived for the relative dispersion of transit times across a single organ [43] for the systemic circulation, the systemic transit time dispersion

14 $${RD}_{C}^{2}={RD}_{B}^{2}+{\left(\frac{1}{1+{V}_{B}/{V}_{T}}\right)}^{2}\frac{Q}{{f}_{u}PS}\left({RD}_{T}^{2}+1\right)$$

is determined by intravascular mixing ${RD}_{B}^{2}$, ie the relative dispersion of circulatory transit time distribution of the intravascular marker and a second term describing tissue distribution kinetics with relative dispersion of tissue residence times ${RD}_{T}^{2}$ [43]. Since all systemic organs were lumped into one subsystem, *V*_*B*_*,V*_*T*_ and *f*_*u*_*PS* are the apparent parameters for the systemic circulation. Although intratissue distribution occurs in reality not instantaneously (${RD}_{T}^{2}$ > 1), a well mixed extravascular space (${RD}_{T}^{2}$ = 1) can be assumed for simplicity. Note that ${RD}_{C}^{2}$> 1 follows from the condition ${RD}_{D}^{2}$> 1 (Eqs. 11 and 13). Although Eq. 14 represents a gross oversimplification of the real multi-organ structure, it may be helpful in explaining the main factors determining *CL*_*D*_, intravascular mixing and blood-tissue exchange. Note that using circulatory models, ${RD}_{C}^{2}$ can be estimated directly and Eq. 14 is only used to interpret the result [23].

To understand the role of ${RD}_{B}^{2}$ in defining ${RD}_{C}^{2}$, it can be derived using a minimal PBPK model; with *n* organs of the systemic circulation arranged in parallel and assuming the organs/tissues as well- mixed subsystems (where ${RD}_{C}^{2}{=RD}_{B}^{2}$) one obtains [33]

15 $${RD}_{B}^{2}=2\sum_{i=1}^{n}\frac{{QV}_{i}^{2}}{{{Q}_{i}V}_{B}^{2}}-1$$

Equation 15 shows that ${RD}_{B}^{2}$ is dependent on flow heterogeneity in the systemic circulation. Since Eq. 15 gives ${RD}_{B}^{2}$=1 for a well-mixed whole body system (*n* = 1), *CL*_*D*_ → ∞ is predicted by Eq. 13 for ${RD}_{C}^{2}$→ 1 (*f*_*u*_*PS* → ∞) in accordance with the limiting case ${RD}_{D}^{2}$ → 1 (monoexponential disposition, Eq. 11). That a two-compartment recirculatory pharmacokinetic model [2, 44] is not capable of predicting the dependence of *CL*_*D*_ on *Q* becomes clear from Eqs. 13 and 14 for ${RD}_{B}^{2}=1$

16 $${CL}_{D}={f}_{u}PS{\left(1+{V}_{B}/{V}_{T}\right)}^{2}$$

Which, as expected is structural identical to *CL*_*D*_ obtained for the two-compartment model (Eq. 6) for ${f}_{u}PS={CL}_{01},{V}_{B}={V}_{0}$ and ${V}_{T}={V}_{1}$.

Although Eq. 14 can be easily extended to *n* organs in parallel [38]

17 $${RD}_{C}^{2}=\sum_{i=1}^{n}\frac{{QV}_{i}^{2}}{{Q}_{i}{V}_{ss}^{2}}\left({RD}_{Ci}^{2}+1\right)-1$$

where ${RD}_{Ci}^{2}$ is given by Eq. 14 with ${RD}_{Bi}^{2}$ ${V}_{B,i}$, ${V}_{T,i}$ and ${f}_{u}{PS}_{i}$, the application of Eq. 17 is not feasible in the present context as discussed above for the PBPK model.
